# Supplementary material for: Recombinant Spider Silk Fiber with High Dimensional Stability in Water and Its NMR Characterization
Source: Molecules. 2022 Dec 2;27(23):8479. doi: 10.3390/molecules27238479 (PMC9739919; doi:10.3390/molecules27238479)
Supplement: Supplementary file 1 [file molecules-27-08479-s001.zip › molecules-1992391-supplementary.pdf]

Supplementary Data

Recombinant spider silk fiber with high dimensional stability in water and their NMR characterization

Tetsuo Asakura[1]*, Hironori Matsuda[1], Akira Naito[1], Hideyasu Okamura[2], Yu Suzuki[2] and Yunosuke Abe[3]

1 Department of Biotechnology. Tokyo University of Agriculture and Technology. 2-24-16 Nakacho, Koganei, Tokyo 184-8588. Japan.
2 Department of Applied Chemistry and Biotechnology, Graduate School of Engineering, University of Fukui, 3-9-1, Bunkyo, Fukui-shi, Fukui 9108507, Japan
3 Spiber Inc., 234-1 Mizukami, Kakuganji, Tsuruoka, Yamagata 997-0052, Japan

Figure S1. Primary structure and amino acid compositions (%) of recombinant spider silk protein, RSP based on the primary sequence of *Araneus diadematus* silk. Each amino acid was colored in G(black), Q(violet), A(red), P(green), S(blue), Y(orange) and others (grey).

Figure S2. Primary structure and amino acid compositions (%) of recombinant spider silk protein (RSSP(QQQ) : Mw＝210 kDa) based on the primary sequence of *Araneus diadematus* silk. Polyalanine-rich sequences were colored red and others black.

Table S1. ^{13}C solution NMR chemical shifts (ppm) of RSSP(VLI) in formic acid.

MHHHHHHHHHHSSGSSLEVLFQGPARA

GSGQQ(GPGQQ)$_3$GPYGPG ASAAAAAA GGYGP

GSGQQGPSQQGPGQQGPGGQGPYGPG ASAAAAAA GGYGP

GSGQQGPGGQGPYGPG SSAAAAAA GGNGP

GSGQQGAGQQGPGQQGPG ASAAAAAA GGYGP

GSGQQGPGQQGPGGQGPYGPG ASAAAAAA GGYGP

GSGQGPGQQGPGGQGPYGPG ASAAAAAA GGYGP

GSGQQ(GPGQQ)$_2$GPGGQGPYGPG ASAAAAAA GGYGP

GYGQQGPGQQGPGGQGPYGPG ASAASAAS GGYGP

GSGQQGPGQQGPGGQGPYGPG ASAAAAAA GGYGP

GSGQQ(GPGQQ)$_3$GPGGQGPYGPG ASAAAAAA GGYGP

GSGQQ(GPGQQ)$_8$GPGGQGAYGPG ASAAAGAA GGYGP

GSGQQ(GPGQQ)$_7$GPYGPG ASAAAAAA GGYGP

GSGQQ(GPGQQ)$_2$GPGGQGPYGPG AASAA

I	G	Q	A	P	S	Y
II	37	19	16	15	6	4

I: Amino acid residue II: Composition (%)

Figure S1. Primary structure and amino acid compositions (%) of recombinant spider silk protein, RSP based on the primary sequence of *Araneus diadematus* silk. Each amino acid was colored in G(black), Q(violet), A(red), P(green), S(blue), Y(orange) and others (grey).

MHHHHHHSSGSSGPGQQGPYGPGASAAAAA
GQNGPGSGQQGPGQSGQYGPGQQGPGQQGPGSSAAAAA
GPGQYGPGQQGPSASAAAAA
GPGSGQQGPGASGQYGPGQQGPGQQGPGSSAAAAA
GQYGSGPGQQGPYGSAAAAA
GPGSGQYGQGPYGPGASGPGQYGPGQQGPSASAAAAA
GSGQQGPGQYGPYASAAAAA
GQYGSGPGQQGPYGPGQSGSGQQGPGQQGPYASAAAAA
GPGQQGPYGPGSSAAAAA
GQYGYGPGQQGPYGPGASGQNGPGSGQYGPGQQGPGQSAAAAA
GPGQQGPYGPGASAAAAA
GQYGPGQQGPGQYGPGSSGPGQQGPYGPGSSAAAAA
GQYGPGQQGPYGPGQSAAAAA
GQYQQGPGQQGPYGPGASGPGQQGPYGPGASAAAAA
GPGQYGPGQQGPSASAAAAA
GQYGSGPGQYGPYGPGQSGPGSGQQGQGPYGPGASAAAAA
GQYGPGQQGPYGPGQSAAAAA
GPGSGQYGPGASGQNGPGSGQYGPGQQGPGQSAAAAA
GQYQQGPGQQGPYGPGASAAAAA
GQYGSGPGQQGPYGPGQSGSGQQGPGQQGPYASAAAAA
GPGSGQQGPGASGQQGPYGPGASAAAAA
GQNGPGSGQQGPGQSGQYGPGQQGPGQQGPGSSAAAAA
GPGQYGPGQQGPSASAAAAA
GPGSGQQGPGASGQYGPGQQGPGQQGPGSSAAAAA
GQYGSGPGQQGPYGSAAAAA
GPGSGQYGQGPYGPGASGPGQYGPGQQGPSASAAAAA
GSGQQGPGQYGPYASAAAAA
GQYGSGPGQQGPYGPGQSGSGQQGPGQQGPYASAAAAA
GPGQQGPYGPGSSAAAAA
GQYGYGPGQQGPYGPGASGQNGPGSGQYGPGQQGPGQSAAAAA
GPGQQGPYGPGASAAAAA
GQYGPGQQGPGQYGPGSSGPGQQGPYGPGSSAAAAA
GQYGPGQQGPYGPGQSAAAAA
GQYQQGPGQQGPYGPGASGPGQQGPYGPGASAAAAA
GPGQYGPGQQGPSASAAAAA
GQYGSGPGQYGPYGPGQSGPGSGQQGQGPYGPGASAAAAA
GQYGPGQQGPYGPGQSAAAAA
GPGSGQYGPGASGQNGPGSGQYGPGQQGPGQSAAAAA
GQYQQGPGQQGPYGPGASAAAAA
GQYGSGPGQQGPYGPGQSGSGQQGPGQQGPYASAAAAA
GPGSGQQGPGASGQQGPYGPGASAAAAA
GQNGPGSGQQGPGQSGQYGPGQQGPGQQGPGSSAAAAA
GQYGYGPGQQGPYGPGASGQNGPGSGQYGPGQQGPGQSAAAAA
GPGQQGPYGPGASAAAAA
GQYGPGQQGPGQYGPGSSGPGQQGPYGPGSSAAAAA
GQYGPGQQGPYGPGQSAAAAA
GQYQQGPGQQGPYGPGASGPGQQGPYGPGASAAAAA
GPGQYGPGQQGPSASAAAAA
GQYGSGPGQYGPYGPGQSGPGSGQQGQGPYGPGASAAAAA
GQYGPGQQGPYGPGQSAAAAA
GPGSGQYGPGASGQNGPGSGQYGPGQQGPGQSAAAAA
GQYQQGPGQQGPYGPGASAAAAA
GQYGSGPGQQGPYGPGQSGSGQQGPGQQGPYASAAAAA
GPGSGQQGPGASGQQGPYGPGASAAAAA
GQNGPGSGQQGPGQSGQYGPGQQGPGQQGPGSSAAAAA
GPGQYGPGQQGPSASAAAAA
GPGSGQQGPGASGQYGPGQQGPGQQGPGSSAAAAA
GQYGSGPGQQGPYGSAAAAA
GPGSGQYGQGPYGPGASGPGQYGPGQQGPSASAAAAA
GSGQQGPGQYGPYASAAAAA
GQYGSGPGQQGPYGPGQSGSGQQGPGQQGPYASAAAAA
GPGQQGPYGPGSSAAAAA
GQYGYGPGQQGPYGPGASGQNGPGSGQYGPGQQGPGQSAAAAA
GPGQQGPYGPGASAAAAA
GQYGPGQQGPGQYGPGSSGPGQQGPYGPGSSAAAAA
GQYGPGQQGPYGPGQSAAAAA
GQYQQGPGQQGPYGPGASGPGQQGPYGPGASAAAAA
GPGQYGPGQQGPSASAAAAA
GQYGSGPGQYGPYGPGQSGPGSGQQGQGPYGPGASAAAAA
GQYGPGQQGPYGPGQSAAAAA
GPGSGQYGPGASGQNGPGSGQYGPGQQGPGQSAAAAA
GQYQQGPGQQGPYGPGASAAAAA
GQYGSGPGQQGPYGPGQSGSGQQGPGQQGPYASAAAAA
GPGSGQQGSSVDKLAAALEHHHHHH

	G	Q	A	P	S	Y
(%)	31	17	20	14	9	7

Figure S2. Amino acid sequence and compositions (%) of recombinant spider silk protein (RSSP : Mw＝210 kDa) based on the primary sequence of *Araneus diadematus* silk. Polyalanine-rich sequences were colored in red and others in black.

Table S1. ^{13}C solution NMR chemical shifts (ppm) of RSSP(VLI) in formic acid.

	Cα	Cβ	Cγ	Cδ	Cε	Cζ
Gly	44.8					
Ala	51.9	18.3				
Pro	62.9	31.2	26.2	49.1		
Ser	54.4[a)]	64.6[b)]				
Tyr	56.8	38.4	129.8	132.3	117.6	156.4
Val	61.6	32.3	20.3, 19.3			
Leu	54.4	42.1	26.2	24.0, 22.6		
Ile	60.5	38.4	26.2, 16.4	11.9		

a) and b) peaks were assigned to the Cα and Cβ peaks of formylated Ser residue, respectively.
